# Modelling and Optimization Studies on a Novel Lipase Production by *Staphylococcus arlettae* through Submerged Fermentation

**DOI:** 10.1155/2013/353954

**Published:** 2013-12-19

**Authors:** Mamta Chauhan, Rajinder Singh Chauhan, Vijay Kumar Garlapati

**Affiliations:** Bioprocess Engineering Laboratory, Department of Biotechnology and Bioinformatics, Jaypee University of Information Technology, Waknaghat, Himachal Pradesh 173 234, India

## Abstract

Microbial enzymes from extremophilic regions such as hot spring serve as an important source of various stable and valuable industrial enzymes. The present paper encompasses the modeling and optimization approach for production of halophilic, solvent, tolerant, and alkaline lipase from *Staphylococcus arlettae* through response surface methodology integrated nature inspired genetic algorithm. Response surface model based on central composite design has been developed by considering the individual and interaction effects of fermentation conditions on lipase production through submerged fermentation. The validated input space of response surface model (with *R* ^2^ value of 96.6%) has been utilized for optimization through genetic algorithm. An optimum lipase yield of 6.5 U/mL has been obtained using binary coded genetic algorithm predicted conditions of 9.39% inoculum with the oil concentration of 10.285% in 2.99 hrs using pH of 7.32 at 38.8°C. This outcome could contribute to introducing this extremophilic lipase (halophilic, solvent, and tolerant) to industrial biotechnology sector and will be a probable choice for different food, detergent, chemical, and pharmaceutical industries. The present work also demonstrated the feasibility of statistical design tools integration with computational tools for optimization of fermentation conditions for maximum lipase production.

## 1. Introduction

Hydrolases particularly lipases (triacylglycerol acylhydrolases, EC 3.1.1.3) from extremophilic microorganisms are experiencing a growing demand, due to their versatile catalytic activities (regio- and enantioselectivity) coupled multifold industrial applications [1]. Among different sources, microbial lipases have gained special industrial attention due to their stability, selectivity, broad substrate specificity, and their cost-effective production. The extracellular bacterial lipases are of considerable commercial importance, due to their substrate specificity, their ability to function in extreme environments, and their bulk production being much easier. Currently bacterial lipases are of great demand because they tend to have neutral or alkaline pH optima and are often thermostable [2, 3].

Lipases from extremophiles are capable of functioning in presence of salts, oxidizing agents, and organic solvents and can withstand the harsh industrial conditions which may permit their use in some specialized industrial applications, such as novel substrates catalysis reactions [4]. Production of lipases through submerged fermentation (SmF) avoids the unwanted metabolites production (usually produced under solid state fermentation) which facilitates easier downstream processing of lipases. Bacterial lipases are mostly extracellular and are greatly influenced by nutritional and physicochemical factors [5–7]. The usual cumbersome variable at a time approach (OVAT) of selecting fermentation conditions for enzyme production fails to give interaction effects of independent variables on the final production yield of enzymes. Response surface methodology (RSM) is a statistical coupled mathematical tool, in which a response of interest is influenced by several variables and the objective is to optimize this response and generates a mathematical model that describes the process by taking individual and interaction effects of the process variables [8]. Several researchers acknowledged the modeling efficiency of RSM for different industrial enzyme production along with the bacterial lipases production from *Bacillus* and *Burkholderia* sp. [9]. Genetic algorithm (GA) is a powerful stochastic search and optimization technique which works on “Survival of Fittest” concept of Darwinian Evolution, which has received considerable attention and replaced the gradient based optimization approaches [10]. It can be used to optimize fermentation conditions without the need of statistical designs and empirical models due to its flexibility in selection of objective function and constraints. The successful utilization of RSM integration with GA for enzyme production has been acknowledged in case of lipase production from *Staphylococcus xylosus* [11] and *Geobacillus* sp. strain ARM [12]. Having a multifold industrial application, a continuous search for extremophilic lipases is underway which can withstand the harsh industrial process conditions. Hence, in the present investigation we have utilized RSM integrated GA based approach for optimization of lipase production by extremophilic [13] *S. arlettae* through SmF for enhanced lipase yield.

## 2. Materials and Methods

### 2.1. Microorganism and Inoculum Preparation

The strain* Staphylococcus arlettae *JPBW-1, previously isolated from rock salt mine Darang Mandi (Himachal Pradesh, India) and identified as a lipase producer, was maintained on the slants of Luria Agar and subculturing was done on every week to maintain its viability. *Staphylococcus arlettae* JPBW-1 was cultivated in LB medium at 35°C for 2 days for spore production.

### 2.2. Chemicals

*p*-nitrophenyl palmitate (Sigma-Aldrich, USA) and LB Broth, Miller (Merck, India) were used for the present study. All other solvents and reagents were either of HPLC grade or AR grade and were obtained from Merck.

### 2.3. Lipase Production through SmF

SmF was carried out by seeding the spore suspension (5 mL) in Erlenmeyer flasks (250 mL) containing 50 mL of the L.B medium, supplemented with soyabean oil (12% v/v). The flasks were incubated at 35°C under agitation (100 rpm) for 3 h. After incubation the fermentation medium was harvested by centrifugation at 6314 ×g for 10 min at 4°C. The supernatant was collected and subjected to estimate the lipase activity.

### 2.4. Lipase Assay

The lipase activity was evaluated spectrophotometrically by measuring *p-*nitrophenol produced by hydrolysis of *p*-nitrophenyl palmitate at 410 nm [14]. One unit (U) of lipase activity was expressed as the amount of enzyme that liberates one micromole of *p*-nitrophenol released per minute under the assay conditions.

### 2.5. Modeling through RSM

RSM is a combination of mathematical and statistical techniques for empirical model building and optimization, which examines the relationships between one or more response parameters and a set of experimental input parameters. This model is only an approximation, but it is used extensively because such a model is easy to estimate and apply, even when little is known about the process. RSM had been used not only for optimization of culture parameters in the fermentation process but also for studying the combined effects of medium components [15].

#### 2.5.1. Selection of Process Parameters for Central Composite Design

Production of lipase through SmF mainly depends on fermentation process variables, namely, temperature (30–40°C), oil concentration (10–14), inoculum size (8–12%), pH (7–9), and incubation time (2–4 h). In the present study based on the central composite design of RSM, design of experiments (DOE) was planned and performed for developing a polynomial response surface model after considering the above-mentioned fermentation variables at three levels.

#### 2.5.2. Statistical Analysis

Based on the one variable at time approach experimental results coupled with literature survey and prior experience in statistical modeling, in the present study, CCD was used by taking five variables at three levels. Nonlinear regression analysis was carried out based on the data collected as per CCD (Table 1) planning for response, namely, lipase activity using MINITAB 14 software which resulted in a second-order polynomial equation.

The coefficient of the nonlinear regression model can be determined using the method of least squares. The effect of the parameters and their interaction terms on the response have been studied by conducting the significance tests and analysis of variance (ANOVA) has been carried out on each response to check the adequacy of the model. The detailed analysis of the effect of parameters and their interactions on the response were also done through the surface plots using MINITAB 14 software.

### 2.6. Artificial Intelligence Based Binary Coded GA Optimization Approach

Optimization is described as the simulation performed aiming to maximize a certain process objective. The search for the desired optimum is usually done using mathematical algorithms. Generally in case of optimization, the problem of interest must be formulated as a mathematical model which describes the system and its performance. The simulation of the process with a mathematical model facilitates the process optimization against highly expensive experiments, predicting process results for any set of decision variables. The simplicity, robustness, and higher convergence rates in lesser computational time account for their popularity in solving the complex, nonlinear problems. These algorithms differ with traditional and gradient based approaches, in searching a population of points in parallel not just a single point and utilizing the probabilistic transition rules instead of deterministic ones. In this context, the present work aims to optimize the nonlinear RSM model of lipase production using artificial intelligence based GA approach. GA is the most popular evolutionary algorithm (EA) which mimics the principle of natural evolution. In GA, the optimization search proceeds through three operators, namely, reproduction, crossover, and mutation [16]. The reproduction (selection) operator selects good strings in a population and forms mating pool. The chromosomes are copied based on their fitness value. No new strings are produced in this operation. Crossover operation generates a child chromosome by exchanging some portion of the strings (chosen randomly) with string of another chromosome in the mating pool using a crossover probability (*P*_*c*_). If the child chromosome is less fit than the parent chromosome, then it will slowly die in the subsequent generation. Mutation was the last operation of GA optimization and used further to perturb the child vector using mutation probability (*P*_*m*_). It alters the string locally to create a better string and to create a point in the neighborhood of the current point, thereby achieving a local search and maintaining the diversity in the population. The entire process is repeated till some termination criterion is met [10]. The mechanics of GA is simply involving coping of the strings. This new population is further evaluated and tested for some termination criteria. In the present study, an attempt has been made to maximize the lipase activity of *S. arlettae *JPBW-1 using binary coded GA by utilizing the input space of the developed RSM model of lipase production through SmF. Taking ten bits for one variable, 50 bits (five input variables) were used to represent a GA string. Based on the concept of duality, the maximization problem is converted to minimization problem. This simulation has been executed through C program.

## 3. Results and Discussion

### 3.1. Modeling Studies through RSM and Statistical Analysis

Most lipases are inducible enzymes and addition of oils proved to enhance lipase activity [17]. RSM is a successive exploratory approach which allows the establishment of the relationship between multiple variables with obtained responses more efficiently than traditional design [18]. The process variables of SmF, that is, incubation temperature, pH, incubation time, inducer concentration (soybean oil %), and inoculum size (%) have been selected as input variables and experiments have been executed based on CCD for developing a second order polynomial response surface model for lipase production by *S. arlettae* (Table 1) using the experimental knowledge of one variable at a time approach for taking the range of each variable.

These experiments were performed in triplicate and lipase activity (*L*_*a*_) of *S. arlettae *JPBW-1 has been expressed as a nonlinear function of the input process parameters in coded form as follows:

(1) La=3.52179−0.01167X1+0.11833X2−0.10222X3+0.51389X4+0.01222X5−0.18187X1X2−0.28062X1X3+0.04188X1X4+0.04188X1X5+0.32312X2X3+0.08312X2X4+0.10313X2X5−0.01062X3X4−0.03562X3X5−0.15813X4X5−0.07564X12+1.57436X22−0.70064X32+0.48436X42−0.56064X52,

where *X*_1_, *X*_2_, *X*_3_, *X*_4_, and *X*_5_ represent temperature, oil concentration, inoculum size, pH, and incubation time, respectively.

**Figure 1:**
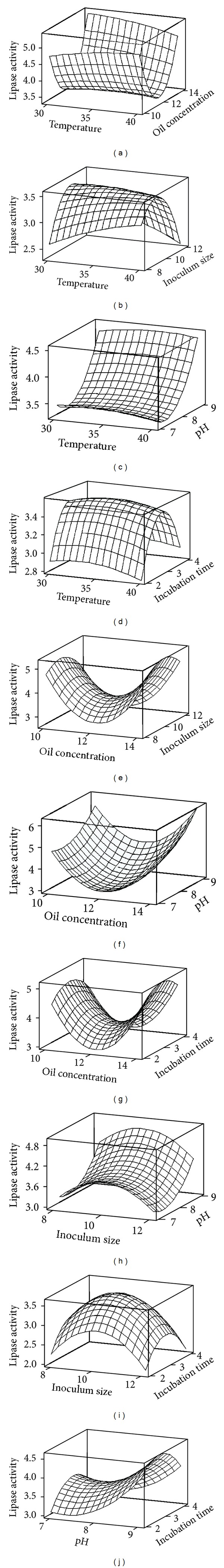
Surface plots of lipase activity with: (a) temperature and oil concentration, (b) temperature and inoculum size, (c) temperature and pH, (d) temperature and incubation time, (e) oil concentration and inoculum size, (f) oil concentration and pH, (g) oil concentration and incubation time, (h) inoculum size and pH, (i) inoculum size and incubation time, and (j) pH and incubation time.

Based on the significance test results (Table 2), the *P* values of *X*_4_, *X*_2_^2^, *X*_3_^2^, *X*_4_^2^, *X*_1_ *X*_2_, *X*_1_ *X*_3_, *X*_2_ *X*_3_, *X*_4_ *X*_5_, and *X*_5_^2^ (found to be less than 0.05, considering 95% (*a* = 0.05) as a level of confidence) are considered as significant terms with impact final lipase activity. The *P* value of the factors *X*_1_, *X*_2_, *X*_5_, and *X*_2_ *X*_5_ is found to be more than the confidence level (0.05) but their square terms *P* values are found to be less than the confidence level indicating their nonlinear relationship with the response, lipase activity. The significant contribution of linear, square, and interaction terms towards the response, lipase activity, has been revealed through ANOVA results (Table 3), where the *P* values of all the terms were found to be less than the significance level *α* = 0.05. The coefficient of multiple regression (*R* ^2^) was seen to be equal to 96.6% which shows the developed model is an adequate predictor of the experimental conditions and confirmed that the selected SmF process variables significantly influence lipase yield [8]. Moreover, *R* ^2^ was found to be in reasonable agreement with adjusted *R* ^2^ (90.5%). The three dimensional response surfaces were plotted to study the interaction among the various factors selected which was found to be its nonlinear relationship with the response, lipase activity (Figure 1). The interaction effect of temperature with oil concentration and pH on lipase activity has been shown in Figures 1(a) and 1(c). In both cases higher lipase activity has been observed nearby high levels (+1) of temperature, oil concentration, and pH. Figures 1(b) and 1(d) illustrate cumulative effect of temperature inoculums size and inoculation time, respectively. Initially, lipase activity (U/mL) increases with time till the maximum value and then again starts decreasing. Interaction of oil concentration with inoculums size (Figure 1(e)) and incubation time (Figure 1(g)) and pH with incubation time (Figure 1(j)) on lipase activity follows the same pattern, where higher lipase activities have been noticed nearby high levels (+1) of oil concentration with inoculums size and incubation time and pH with incubation time compared to the respective low (−1) and middle (0) levels. Interaction effect of oil concentration and Ph with lipase activity has been depicted in Figure 1(f). When operating at high oil concentration and Ph, lipase activity seems to be higher when compared with the initial and middle levels of oil concentration and pH. Therefore, the interaction effect of oil concentration and pH was tested as an important variable to enhance the lipase activity in SmF.  Figure 1(h) showed the interactive effect of inoculums size and Ph on lipase activity. It revealed that the higher lipase activity has been observed at higher Ph at all inoculums sizes. As shown in Figure 1(i), lipase activity was influenced by inoculums size and incubation time. The maximum lipase activity has been achieved when inoculums size and incubation time near their zero levels. The nonlinear relationship of fermentation process variables, namely, inoculum concentration and temperature on lipase yield from *Geobacillus thermoleovorans* CCR11 has been also found through the RSM approach [19]. Significant and combined effects of polydimethylsiloxane (PDMS) and oxygen volumetric mass transfer coefficient through RSM have been acknowledged by Rech et al. [20] for lipase production by *Staphylococcus warneri* EX17.

### 3.2. GA Based Optimization

**Figure 2:**
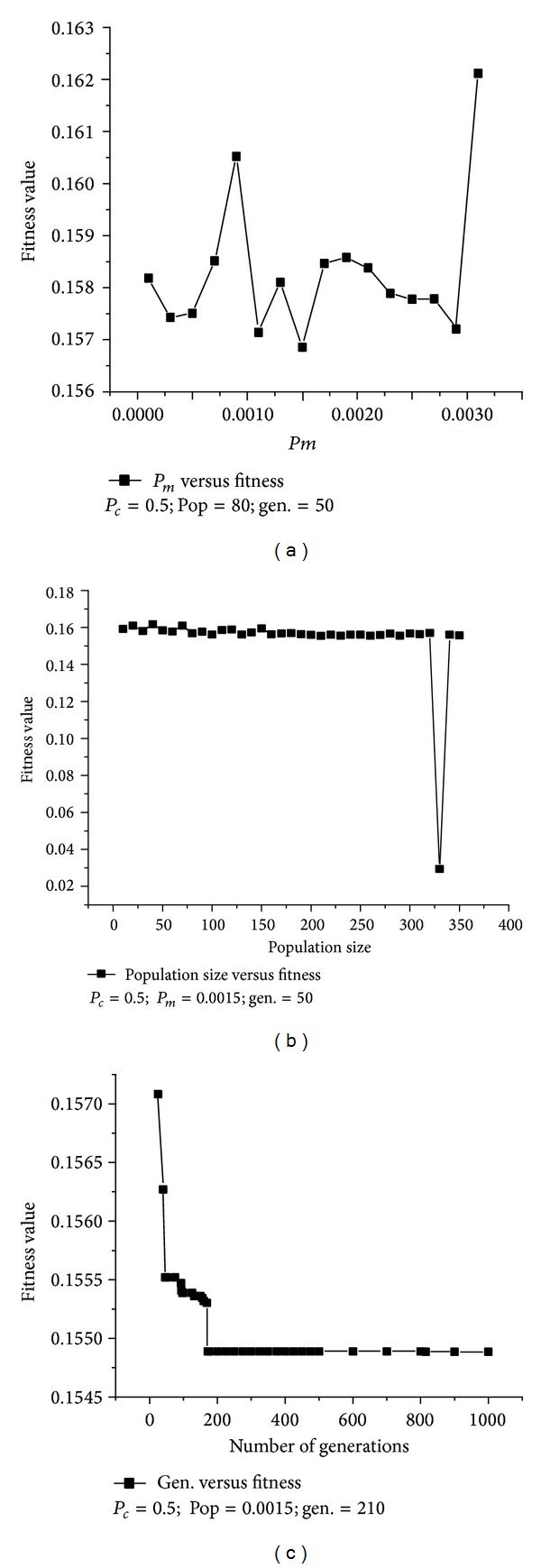
Results of parametric study of GA (a) mutation probability (*P*_*m*_) versus fitness, (b) population size versus fitness, and (c) maximum number of generations versus fitness.

Genetic algorithm is a stochastic optimization technique that searches for an optimal value of a complex objective function and is used to solve complicated optimization problems by simulation or mimicking a natural evolution process [21]. The application of genetic algorithms in bioprocess optimization had been reported by researches which are more flexible tool used here for minimization of reaction time while maximizing product concentration [22]. The selection of population size, number of generations, mutation probability, and crossover mechanism plays an important role in exploring the input space of the problem of interest by GA. In the present study, RSM model of lipase extraction is posed as an optimization problem for maximizing the lipase activity. A systematic study was conducted to determine the GA parameters responsible for optimal value of lipase activity. The results of parametric study of GA have been shown in Figure 2 for searching optimal fermentation process variables to predict the final lipase yield. Figure 2 shows the parametric analysis of *P*_*m*_ (0.001–0.0031), population size (10–350), and maximum generations (25–1000) versus fitness value; from this analysis we have selected the optimum values of *P*_*m*_, population size, and maximum generation number at which optimum fitness value has been noticed one by one. In the present study, tournament selection of size two, uniform crossover probability (*P*_*c*_) of 0.5, bitwise mutational probability (*P*_*m*_) of 0.0015, population size of 210, and maximum number of generations of 815 were employed in search of optimal values of the lipase extraction from the fermented broth for enhanced lipase activity. These optimized parametric parameters (*P*_*c*_, *P*_*m*_, pop. Size, and max. gen) have been utilized for final run of binary coded GA, which results in 50-bit length string that represents the five optimized input variables (10 bits for each variable) of SmF. After decoding these strings to real values through linear mapping, the optimum values of process parameters were seen to be equal to 38.82°C, 10.285%, 9.392%, 7.32, and 2.995 h for fermentation variables such as temperature, oil concentration, inoculums size, pH, and incubation time, respectively. Moreover, the maximum value of lipase activity for* S. arlettae *JPBW-1 was found to be equal to 6.456375 U/mL.

#### 3.2.1. Experimental Validation of GA Proposed Optimization Results

To confirm these results, lipase production was conducted with the optimum levels of the significant factors representing the maximum lipase yield 6.45 U/mL. The lipase yield improved about 1.8-fold than the one at a time approach for optimization of lipase production in which the lipase yield was 3.54 U/mL. The significant correlation between predicted and observed values of lipase yield in these experiments justified the validity of the response model and the existence of an optimum point. Among the various artificial intelligence techniques, genetic algorithms, a powerful stochastic search and optimization technique, have received considerable attention. Genetic algorithms can be used to optimize fermentation conditions without the need of statistical designs and empirical models. Implementation of a GA for multiobjective experimental optimization was recently demonstrated [23] and, thus, offers the chance for further reduction of the experimental effort. Ebrahimpour et al. [12] have also utilized artificial intelligence techniques for enhanced lipase production from a newly isolated thermophilic Geobacillus sp. strain ARM. The better search criteria of GAs for optimal conditions have been acknowledged in case of polyhydroxybutyrate (PHB) production by *Azohydromonas lata* MTCC 2311 [24] and in laccase mediated biodegradation of 2,4-dichlorophenol [25]. Moreover, preliminary results show that lipase from *Staphylococcus arlettae *JPBW-1 showed stability in presence of salt (up to 30% NaCl) and organic solvents (up 30% benzene, xylene, *n*-hexane, and toluene) and has ability to work in extreme conditions of temperature (up to 70°C) and pH (8–12) [13].

## 4. Conclusion

To summarize, this study presents evaluation of RSM integrated GA in modeling and optimization of lipolytic activity production from *S. arlettae.* A second order polynomial response surface model has been developed successfully and utilized in search of optimal conditions for lipase production through SmF using binary coded GA. The optimum fermentation conditions obtained for the synthesis of lipase from *S. arlettae* were 38.8°C, oil concentration 10.2%, inoculum volume 9.3%, pH 7.32, and incubation time 3 h for obtaining a maximum lipase activity of 6.45 U/mL. An overall 1.8-fold increase in lipase activity was achieved after fermentation variables optimization, following the statistical approach. The high tolerance of this lipolytic enzyme under extreme conditions will make it an enzyme of choice for many industries and considered to be a good candidate for its viability for commercialization.

## Figures and Tables

**Table 1:** Central composite design with the experimental, predicted responses and its *R*-studentized residuals.

Run	Input parameters					Response, La (U/mL)		*R*-studentized residual
	Temp.^a^ (°C)	OC^b^ (%)	IS^c^ (%)	pH	IT^d^ (h)	Exp.	Predict.	
	(*X*_1_)	(*X*_2_)	(*X*_3_)	(*X*_4_)	(*X*_5_)			
1	35	12	10	8	4	3.06	2.973	0.479
2	30	14	12	7	4	4.62	4.708	−2.195
3	30	10	12	9	4	3.86	3.862	−0.042
4	30	10	8	7	4	3.74	3.761	−0.437
5	30	14	12	9	2	5.68	5.722	−0.884
6	35	12	10	8	3	3.26	3.528	−1.111
7	35	10	10	8	3	4.92	4.978	−0.318
8	40	14	8	9	2	4.87	4.843	0.561
9	40	14	8	7	4	3.91	3.929	−0.402
10	35	12	10	8	3	3.27	3.522	−1.064
11	35	12	8	8	3	2.90	2.923	−0.128
12	40	10	8	9	4	5.38	5.313	1.509
13	30	12	10	8	3	3.71	3.458	1.533
14	40	10	12	9	2	4.33	4.294	0.748
15	35	12	10	8	3	3.26	3.522	−1.111
16	35	12	10	8	2	3.86	3.522	1.491
17	30	10	8	9	3	4.78	4.754	0.525
18	35	12	10	9	2	4.42	4.520	−0.556
19	30	14	8	7	3	3.16	3.221	−1.351
20	40	12	8	7	2	3.37	3.434	−0.355
21	30	10	10	8	4	3.39	3.442	−1.338
22	40	10	12	7	3	3.19	3.201	−0.224
23	35	14	12	7	4	5.46	5.214	1.484
24	40	14	10	8	2	4.70	4.700	−0.009
25	40	14	12	9	3	3.15	3.200	−1.093
26	35	12	12	7	4	2.93	2.719	1.240
27	30	14	12	8	2	4.56	4.577	−0.219
28	35	12	8	9	3	3.05	2.949	0.561
29	35	12	10	8	3	3.78	3.492	1.815
30	35	12	10	7	3	3.35	3.522	−0.705
31	35	12	10	8	3	3.38	3.522	−0.577
32	35	12	10	8	3	3.35	3.522	−0.705
33	35	12	10	8	3	3.38	3.522	−0.577

^a^Temperature; ^b^oil concentration; ^c^inoculum size; ^d^incubation time.

**Table 2:** Results of significance test on the nonlinear model coefficients, standard errors, *T* statistics, and *P* values for the lipase activity (coded form).

SI. no.	Standard				
	Terms	Coefficient	Error coefficient	*T*	*P*
1	Constant	3.528	0.071	49.618	0.000
2	*X*_1_	−0.0117	0.058	−0.199	0.846
3	*X*_2_	0.118	0.058	2.021	0.068
4	*X*_3_	−0.102	0.058	−1.746	0.109
5	*X*_4_	0.514	0.058	8.778	0.000
6	*X*_5_	0.012	0.058	0.209	0.838
7	*X*_1_^2^	−0.075	0.158	−0.478	0.642
8	*X*_2_^2^	1.574	0.158	9.944	0.000
9	*X*_3_^2^	−0.707	0.158	−4.425	0.001
10	*X*_4_^2^	0.484	0.158	3.059	0.011
11	*X*_5_^2^	−0.560	0.158	−3.541	0.005
12	*X*_1_ *X*_2_	−0.182	0.062	−2.929	0.014
13	*X*_1_ *X*_3_	−0.287	0.062	−4.520	0.001
14	*X*_1_ *X*_4_	0.049	0.062	0.674	0.514
15	*X*_1_ *X*_5_	0.041	0.062	0.674	0.514
16	*X*_2_ *X*_3_	0.323	0.062	5.204	0.000
17	*X*_2_ *X*_4_	0.083	0.062	1.339	0.208
18	*X*_2_ *X*_5_	0.103	0.062	1.661	0.125
19	*X*_3_ *X*_4_	−0.010	0.062	−0.171	0.867
20	*X*_3_ *X*_5_	−0.035	0.062	−0.574	0.578
21	*X*_4_ *X*_5_	−0.158	0.062	−2.547	0.027

	SS = 0.2484	*R* ^2^ = 96.6%		*R* ^2^ (adj) = 90.5%	

**Table 3:** Results of ANOVA-lipase activity.

Source	DF	Sequential	Adjusted		*F*	P
		SS	SS	MS		
Regression	20	19.5268	19.5268	0.97634	15.83	0.000
Linear	5	5.1987	5.1987	1.03975	16.86	0.000
Square	5	10.1093	10.1093	2.02185	32.78	0.000
Interaction	10	4.2188	4.218	0.42188	6.84	0.002
Residual error	11	0.6785	0.6785	0.06168		
Lack-of-fit	6	0.4080	0.4080	0.06800	1.26	0.410
Pure error	5	0.2705	0.2705	0.05411		

Total	31	20.2053				
